# COVID-19 Outbreak in North Italy: An Overview on Dentistry. A Questionnaire Survey

**DOI:** 10.3390/ijerph17113835

**Published:** 2020-05-28

**Authors:** Maria Grazia Cagetti, Jean Louis Cairoli, Andrea Senna, Guglielmo Campus

**Affiliations:** 1 Department of Biomedical, Surgical and Dental Science, University of Milan, Via Beldiletto 1, I-20142 Milan, Italy; maria.cagetti@unimi.it (M.G.C.); andreasenna75@hotmail.com (A.S.); 2 Private Practitioner, President of the Lombardy Chamber of Dentists, Varese, Italy; jlcairo@gmail.com; 3 Department of Surgery, Microsurgery and Medicine Sciences, School of Dentistry, University of Sassari, Viale San Pietro, I-07100 Sassari, Italy; 4 Department of Restorative, Preventive and Pediatric Dentistry, University of Bern, Freiburgstrasse 7, CH-3010 Bern, Switzerland

**Keywords:** COVID-19, infection, dentist, protective measures, awareness, infection control

## Abstract

This survey assessed the symptoms/signs, protective measures, awareness, and perception levels regarding COVID-19 among dentists in Lombardy, Italy. Moreover, an analysis of the answers gathered in areas with different prevalence of the disease was carried out. All Lombardy’s dentists were sent an online ad hoc questionnaire. The questionnaire was divided into four domains: personal data, precautionary measures (before patient arrival; in the waiting room; in the operating room), awareness, and perception. Three thousand five hundred ninety-nine questionnaires were analyzed. Five hundred two (14.43%) participants had suffered one or more symptoms referable to COVID-19. Thirty-one subjects were positive to the virus SARS-CoV-2 and 16 subjects developed the disease. Only a small number of dentists (*n* = 72, 2.00%) were confident of avoiding infection; dentists working in low COVID-19 prevalence areas were more confident than those working in the Milan area and high prevalence area (61.24%, 61.23%, and 64.29%, *p* < 0.01 respectively). The level of awareness was statistically significantly higher (*p* < 0.01) in the Milan area (71.82%) than in the other areas. This survey demonstrated that dentists in the COVID-19 highest prevalence area, albeit reported to have more symptoms/signs than the rest of the sample, were the ones who adopted several precautionary measures less frequently and were the more confident of avoiding infection.

## 1. Introduction

The coronavirus pandemic has deeply affected the world. Up to 12 May 2020, the total number of confirmed cases has exceeded four million and a half, with more than two hundred eighty thousand deaths. The SARS-CoV-2 human-to-human transmission has been described through airborne droplets or direct contact with cases or with contaminated surfaces [1]. Avoiding close contact (less than 1 m) with people, especially those with respiratory symptoms, is the most important preventive measure to be taken to prevent the spreading of the infection.

In May 2020, Italy is still among European countries with the highest number of Covid-19 cases, now in third place after Spain and the United Kingdom. The majority of cases are concentrated in the Northern part of the country (Lombardy) and held the sad European deaths record [2]. Another dark Italian record is the number of health care workers who were infected or who died as a result of the infection. The official number of infected health workers up to 12 May, 2020, according to the Italian Superior Health Institute, amounted to 21.981 workers [3]. According to the Italian National Federation of the Order of Physicians, Surgeons, and Dentists, instead, the number of deceased physicians up to the 10 May, 2020 amounted to 160 deaths [4], of whom sixteen were dentists. Although patients affected by COVID-19 are not supposed to receive dental treatments, undiagnosed infected subjects without or with very mild symptoms could be eligible for dental treatment in emergency cases. Dental care in Italy is largely provided by private practitioners and mainly financed by patients’ direct payment, or, to a lesser extent, by private insurance schemes. 

The risk of cross-infection in dentistry has been described considerably high [5] since splatters and aerosols produced during routine dental treatments contribute to increased risk [6]. This issue might be a relevant professional hazard when infective agents, such as coronaviruses, are widespread in the population [7]. Dentists and health care professionals working in wards with pneumonia patients are at higher risk of developing infective diseases during their regular activities [8]. Data on the real risk of virus diffusion by dental procedures are urgent since none is available in the literature [8,9]. In a recent paper, the stability of SARS-CoV-2 and SARS-CoV-1 in aerosols and on various surfaces was investigated in experimental conditions, showing that the airborne transmission of SARS-CoV-2 is plausible since the virus can remain viable and infectious in aerosols for hours [9]. Without data on airborne SARS-Cov-2 gained in real dental care situations, operational envelopes and disinfection procedures to face the viral infection are hypothetical.

Well-designed questionnaires are a useful method to easily collect data from participants in studies [10]. Questionnaires to investigate dentists’ knowledge, attitudes, and perceptions regarding viral infection control in the dental environment found in the literature [11,12,13,14] show that awareness and precautionary measures carried out by dentists on patients with a viral infection are not always completely satisfactory. The main aim of this survey was to assess the symptoms/signs, the protective measures, the level of awareness, and perception regarding the COVID-19 outbreak among dentists working in North Italy. The ancillary aim was also to appraise if the answers provided bear resemblance in areas with different prevalence of the disease. 

## 2. Materials and Methods

### 2.1. Development and Building-Up of the Questionnaire

The first bunch of items related to the health situation, risk, and knowledge of an infectious disease was derived from the questionnaire developed for the SARS risk [15]. The authors followed the Stehr-Green scale to build up the questionnaire [16]. The questionnaire was structured into four domains, the first regarded personal data (age, gender, area of living, and working status), the second the health conditions (symptoms/signs relative to the COVID-19 flu), the third the working condition and personal protective equipment (PPE) adopted after the outbreak of the infection, and the fourth the knowledge and the self-perceived risk of infection (Table 1). Among the PPE included in the questionnaire, some, such as the use of sterile gloves, do not have a scientific justification but were deliberately inserted to check whether the answers were selected with the sole logic of demonstrating that any contrast measures regarding the virus had been implemented or whether the equipment adopted was the result of a thoughtful choice.

A preliminary questionnaire was built up and pre-tested on a small group of dentists (*n* = 12); Intraclass Correlation Coefficients (ICC) was run for the test-retest and intra-rater reliability for each item. An ICC value of 0.80 or higher was considered satisfactory. All the items with a value of ICC below 0.80 were discussed by the authors and modified following the preliminary study.

An anonymous online survey (Survey Monkey™, SVMK Inc. San Mateo, CA, US) has been prepared. On the 10th of April, all dentists *n* = 9247 included in the database of the Order of Physicians, Surgeons, and Dentists of Lombardy, 89.79% of all dentists registered in Lombardy, received an email asking their consent to participation in the questionnaire in accordance with applicable privacy laws. All the participants were asked to declare that they have read the privacy policy and voluntarily approve data collection and processing. If they answered No, the questionnaire was automatically closed, and no data were collected. A second reminder was emailed to the non-responders after four days and the last one on the 16th of April. The survey was stopped one week after its beginning.

### 2.2. Data Analysis

Answers to the questionnaire were inserted in Excel™ 2019 for Mac. The data were cleaned and then transferred to STATA16™ (StataCorp LLC, College Station, Texas, USA) for their statistical analysis. The 12 Lombardy provinces were grouped as follows: Milan province, with a COVID-19 prevalence of 0.53%, was considered alone, and provinces where the prevalence of COVID-19 was higher than 0.90% (Cremona, Lodi, Brescia, Bergamo) were grouped together. Provinces with lower prevalence (Varese, Como, Monza, Sondrio, Lecco, Pavia, Mantua) with a mean of 0.44 (data evaluated 24th April) were also grouped together [5]. Absolute and relative frequencies were calculated for each item. Difference in proportion was evaluated with χ^2^ test or Fisher exact test if one cell had a value of less than five. Multiple testing for post hoc estimation was calculated, such as the number of observed frequencies, expected frequencies, percentage, and contribution to the chi-square. The symptoms most frequently reported in the literature (fever, cough, fatigue) were used for a comparison between areas with different COVID-19 prevalence [17]. A *p*-Value less than 0.05 was considered statistically significant. The row data are available as supplementary materials (Table S1). 

## 3. Results

In the pre-test evaluation, only two items showed an ICC below the threshold (i.e., “Which of the following protective equipment did you wear/use?” ICC = 0.73 and “Do you believe that the infection by SARS-CoV-2 is a risk for the dentist?” ICC = 0.78) and, after discussion among the authors, the questions were slightly modified. A total of 9247 invitations were emailed, and 112 (1.21%) were not delivered by the system. After the first dispatch, 65.95% of the emails were opened: 1.32% refused and 41.60% participated in the questionnaire. At the end of the survey, 4308 questionnaires were returned. Three thousand five hundred ninety-nine questionnaires (response rate 39.40%) were analyzed (69.27% males and 30.73% females). A statically significant (*p* < 0.01) predominance of males was observed among dentists who compiled the questionnaire (Table 2).

Thirty-one subjects (0.86% of the dentists whose questionnaires were analyzed) were positive to the virus SARS-CoV-2, and 16 subjects developed the disease. The triage of symptoms/signs related to COVID-19 showed that 474 (13.47%) participants claimed to have suffered one or more symptoms/signs referable to COVID-19.

Among the symptoms/signs (Table 3), the sense of fatigue and fever were the most common (7.63 and 7.21%, respectively), while breath difficulties and conjunctivitis were the less frequent (1.98 and 1.98%, respectively). Almost 10% of the dentists working in area with a high prevalence of COVID-19 reported to suffer or have suffered from three or more symptoms (χ^2^_(6)_ = 63.64 *p* < 0.01 post ad hoc estimation likelihood-ratio χ^2^_(6)_ = 62.12 *p* < 0.01). 

The three main common symptoms from the literature (fever, cough, and fatigue) were statistically highly (χ^2^_(6)_ = 59.20 *p* < 0.01 Post ad hoc estimation Likelihood-ratio χ ^2^_(6)_ = 52.31 *p* < 0.01) reported from dentists working in Milan and the high prevalence area (Table 4).

More than 90% of the responders worked as private dentists and only 242 (6.82%) worked partially or full-time in the National Health System (NHS). Almost half of the dentists continued to work after the outbreak of the disease (21st February). 

Several precautionary measures were adopted by dentists who continued to work after SARS-CoV-2 outbreak; in Table 5, the measures were grouped in (1) measures adopted before the patient’s arrival, (2) measures adopted in the waiting room, and (3) measures adopted in the operating room. Among measures taken before the patient’s arrival, the delay of the appointments to not saturate the waiting room was the most adopted (86.07%). Frequent ventilation of the waiting room (88.98%) and the washing of the operators’ hands before and after each procedure (91.64%) were the most taken measures.

Table 5 reports precautionary measures with more than 80% positive replies, among those of Table 4, stratified by areas with a different prevalence of COVID-19. Statistically significant differences were found for all considered items. The delay of the appointments in order to not saturate the waiting room, the frequent ventilation of the waiting room, and the washing of the operators’ hands before and after each procedure were the items with the higher differences among areas (*p* < 0.01). Surprisingly, dentists from the area with the highest COVID-19 prevalence claimed to have used some virus containment strategies, such as the disinfection of pushbuttons, point of sale (POS), and chairs several times a day, the removal of all disposable protective devices, and disinfection of devices and washing hands, less frequently than dentists who work in the lower prevalence areas (Table 6).

In addition to the PPE commonly used by dentists, such as the use of disposable gloves (93.22%) and surgical masks (74.56%), the use of glasses/visors (91.28%), disposable headsets (63.75%), and facial filters (58.84%) were the equipment most claimed (Table 7).

Only one-third of the dentists reported to have followed a Continuous Educational Course on COVID-19, but 70.49% of the sample believed to have enough knowledge on the disease and the protective measures (data not in tables).

About the risk perception of being infected by SARS-CoV-2 (Table 8), the majority of the dentists (64.50%) replied that the dentistry is a profession at risk; only 2.13% of the dentists claimed to be confident in avoiding the infection and 68.50% believed that in the actual health emergency, the risk of infection transmission during the dental practice is higher than that run in a supermarket.

The same variables mentioned above were stratified by areas with different prevalence of COVID-19 (Table 8). Unlike what could be assumed, even though only a small number of dentists in all areas believe to be confident in avoiding the infection, dentists working in areas with a high COVID-19 prevalence are more confident than those working in a lower prevalence area (61.23% vs 64.29% and 66.41%). Dentists from different areas agree that the risk of infection is higher in the dental setting than in a supermarket, but a statistically significant difference among areas was noted (63.63% in high COVID-19 area, 68.25% in low COVID-19 area, and 71.82 in Milan area (Table 9). 

## 4. Discussion

The present survey was carried out during the period of maximum diffusion of COVID-19 in Europe. Lombardy, situated in Northern Italy, with about 10 million inhabitants (more than one-sixth of Italy’s entire population), is the region with the highest number of SARS-CoV-2 infections and deaths. 

The sample of dentists to whom the questionnaire was emailed includes almost all Lombardy dentists. The response rate was quite low; however, given the high number of questionnaires sent, the sample of responders is high and representative of the Lombardy dentist population.

At the moment in which this paper was written, three papers were available in literature reporting data collected through a questionnaire administered to a sample of dentists investigating different aspects of the COVID-19 in the dental setting [13,14,18]. The first two papers investigated knowledge, attitudes, and practices of dental practitioners regarding COVID-19, one study involving a sample of dentists from different countries and continents and the second involving a sample of dentists from Jordan [13,14,18]. The third study, including a sample of dentists from all over the world, aimed to assess fear and practice modifications related to COVID-19 [18]. None of these studies addressed the health conditions of dentists related to the disease. In the present survey, among the interviewed dentists, the percentage of subjects diagnosed with the new coronavirus (0.86%) is similar to that reported in the population of high COVID-19 prevalence areas. This data could suggest a greater infection diffusion among dentists. However, this finding could be due to a possibly higher participation rate in the questionnaire of subjects infected with the virus or with claimed symptoms/signs. They were reported by a relatively high percentage of dentists (14.43%). Nevertheless, these symptoms/signs may have been caused by other conditions such as seasonal flu, still present in the period of the widespread of SARS-CoV-2. However, the highest prevalence reported by dentists working in the provinces where COVID-19 had spread, such as Bergamo and Cremona, is startling.

Regarding the precautionary measures taken by dentists that continued to work after the outbreak of COVID-19, it is possible to compare these data with those reported in a worldwide taken sample of dentists [19]. Patients’ body temperature before dental treatment was taken by less than a quarter of the Lombardy sample, while this measure was carried out by more than two-thirds of dentists interviewed all over the world. In the same study, considering the use of PPE, the majority of dentists reported to believe that the use of facial filters is a useful habit in the current outbreak, but only a minority claimed to use it. More than half of the Lombardy sample declared to use these PPE. Only a quarter of the international sample of dentists make their patients do a pre-treatment mouth-rinse, while in Lombardy, the majority of dentists use this protective measure on patients. Nevertheless, it is important to note that half of the Lombardy sample reported using chlorhexidine-containing rinse that appears not to be efficient against SARS-CoV-2, and only one-third reported to use a mouth-rinse containing more active compounds [19]. Finally, handwashing before and after each treatment was a habit reported by a high percentage of dentists from both samples. The majority of dentists from both surveys are afraid of getting infected with SARS-CoV-2 in the dental environment.

The use of sterile gloves and gown as well as other PPE included in the present questionnaire do not have a scientific justification in this pandemic situation, as reported above. Regarding the use of gloves, only a small minority of dentists claimed to use sterile gloves, while the use of sterile gowns was reported by about a fifth of the sample. However, it is possible to hypothesize that dentists unprepared for the pandemic used PPE that they already had to protect themselves, albeit knowing that some, such as sterile gloves and gowns, were not necessary to avoid the infection.

Unlike what could be expected, for both preventive measures and self-perceived infection risk related to COVID-19, dentists from the areas with the highest prevalence of the disease seem to be generally less preoccupied: they reported a lower implementation of some of the most frequently adopted preventive measures than their colleagues from areas at low COVID-19 prevalence as well as a lower perception of being infected. The different perception of the risk reported by dentists who live and work in areas with a different prevalence of the disease can be explained by the fact that where many infected people are present, the risk is seen as general, reducing the perception of a higher infection risk at the dental chair, while dentists who live and work in areas with a lower prevalence of the disease consider the occupational risk as higher.

Only one-third of the dentists reported to have followed a Continuous Educational Course on COVID-19, but more than two-thirds believe to have enough knowledge about the new disease. This discrepancy could represent a weakness. Throughout this international health crisis, a large amount of information reaches us every day, involving the circulation of many fake news, which can represent a danger especially in the health context [20].

## 5. Conclusions

In conclusion, this survey gives an insight into the dental profession in one of the European areas where COVID-19 has caused the greatest number of deaths in proportion to the number of inhabitants. A quite high percentage of the sample reported symptoms attributable to the infection, especially those working in the high prevalence area. However, only 31 of these subjects were diagnosed with COVID-19. Even though the majority of dentists adopted several precautionary measures, recognized as valid by the scientific community, those working in the highest prevalence COVID-19 area reported adopting several measures less frequently than dentists in low prevalence area. The same unexpected finding was disclosed regarding the COVID-19 risk perception: dentists in the highest prevalence area were more confident to avoid the infection than others.

Only one-third of the dentists report to have followed a Continuous Educational Course on COVID-19, but the majority of the sample believes to have enough knowledge on the disease and the protective measures to avoid infection.

## Figures and Tables

**Table 1:** Questionnaire items.

Items	
Gender	male
	female
Age	
Zip Code (living)	
Zip Code (working)	
Working status	Private dentist
	Private/NHS
	NSH
From the start of the COVID-19 you had	No symptoms/signs
	You resulted COVID-19 positive
	You were hospitalized for COVID
	I had one/more symptoms/signs
	Fever
	Cough
	Fatigue
	Short Breath
	Nasal congestion
	Headache
	Rhinorrhea
	Sore throat
	Diffuse pain
	Diarrhea
	Anosmia
	Ageusia
	Conjunctivitis
Only if you work in the NHS, are you currently working?	Yes
	No
From the 21st February	You kept working as usual
	You limited your activity to emergencies
	You have stopped all activities
If you have limited your professional activity to emergencies, when did you start limiting?	Between 21–23 February
	Between 24 February and 1 March
	Between 2–6 March
	Between 7–14 March
	After 14th March
If you have stopped your professional activity, when did this happen?	Between 21–23 February
	Between 24 February and 1 March
	Between 2–6 March
	Between 7–14 March
	After 14th March
If you have continued working after 21st February, which of the following measures have you adopted?	None
	Phone Triage
	Spaced appointments so to not saturate the waiting room
	Deferring therapies in elderly patients, or patients with systemic diseases
	Handle disinfection several times a day
	Disinfection of pushbuttons, Point of sale, chairs several times a day
	Verify the patient’s current health status on access
	Detecting the patient’s body temperature
	Detecting the body temperature of all co-workers and ask to leave to those with a temperature above 37.5 °C.
	Washing the patient’s hands
	Space of at least one meter between patients
	Mask for the patient
	Frequent ventilation of waiting rooms
	Removal of magazines and books from the waiting area
	Storage of coats, bags, and other items outside the operating area
	Pre-operative rinse with mouthwash containing 1% hydrogen peroxide
	Pre-operative rinse with mouthwash containing chlorhexidine 0.12–0.2%
	Pre-operative rinse with mouthwash containing 0.2–1% iodopovidone
	Pre-operative rinse with mouthwash containing alcohol and essential oils
	Pre-operative rinse with mouthwash containing Cetylpyridinium chloride at 0.05–0.10%
	Rinse with diluted mouthwash
	Ventilation of the operating area for at least 10 min after each patient
	Surface disinfection with 70% ethyl alcohol
	Surface disinfection with 0.5% sodium hypochlorite
	Surface disinfection with usual disinfectants containing other active ingredients
	Washing operators’ hands before and after each procedure
	Removal of all disposable protective devices and disinfection of non-disposable devices
Which of the following protective equipment did you wear/use?	Surgical mask
	Filtering facepiece 2 or filtering facepiece 3 masks
	Disposable headset
	Sterile microfiber disposable gown
	Water-repellent, non-wowen fabric TNT disposable gown
	Disposable gown
	Safety glasses or visor
	Sterile disposable gloves
	Disposable gloves
	Rotating instrument with anti-retraction valve
Did you follow a course on Covid-19?	Yes
	No
Do you think that you know enough on COVID-19?	Yes
	No
Do you believe that the infection by SARS-CoV-2 is a risk for the dentist?	Unlikely
	Very unlikely
	Likely
	Very likely
How sure are you that you can avoid becoming infected with SARS-CoV-2 during work activities?	No confident
	Enough confident
	A bit confident
	Confident
In a health emergency situation such as the current one, do you believe that the risk of infection transmission in the dental practice is:	Less than the risk run in a supermarket
	Comparable to the risk run in a supermarket
	Higher than the risk run in a supermarket

**Table 2:** Participants’ distribution by age and gender.

Age Groups	Males *n (%)*	Females *n (%)*	Total *n (%)*
<30 years	180 (5.02)	181 (5.05)	361 (10.07)
31–40 years	350 (9.76)	271 (7.56)	621 (17.32)
41–50 years	401 (11.18)	270 (7.53)	671 (18.71)
51–60 years	692 (19.30)	242 (6.75)	934 (26.05)
>60 years	861 (24.01)	138 (3.85)	999 (27.86)
Total	2493 (69.27)	1106 (30.73)	3599 (100.00)

χ ^2^_(4)_ = 285.48 *p* < 0.01.

**Table 3:** Prevalence of symptoms/signs related to the COVID-19 in the different Lombardy provinces. Percentages were calculated per column.

Milan Area				High Prevalence Area				Low Prevalence Area			
OF	EF	%	C**χ**^2^	OF	EF	%	C**χ**^2^	OF	EF	%	C**χ**^2^
No symptoms											
1072	1067.47	86.80	0.02	721	784.83	79.41	5.19	1221	1161.69	90.86	3.03
One symptom											
38	34.35	3.08	0.39	39	25.26	4.29	7.47	20	25.26	1.50	8.09
Two symptoms											
59	57.73	4.78	0.03	61	42.45	6.72	8.11	43	42.45	3.20	6.26
Three or more symptoms											
66	75.44	5.34	0.14	87	55.46	9.58	17.93	60	55.46	4.46	5.95

χ^2^_(6)_ = 63.64 *p* < 0.01 Post ad hoc estimation Likelihood-ratio χ^2^_(6)_ = 62.12 *p* < 0.01. OF, observed frequency; EF, expected frequency; %, percentage; Cχ^2^, contribution to chi-square.

**Table 4:** Prevalence of the most associated symptoms/signs related to the COVID-19 in the different Lombardy provinces. Percentages were calculated per column.

Symptoms/Signs	Milan Area				High Prevalence Area				Low Prevalence Area			
	OF	EF	%	OF	EF	%	OF	EF	%	OF	EF	%
Fever	17	16.29	11.97	0.03	21	11.98	13.38	6.80	8	17.73	7.84	5.34
Cough	15	19.50	10.57	1.03	26	14.32	16.56	9.52	14	21.20	13.73	2.45
Fatigue	25	21.61	17.60	0.53	23	18.88	16.65	3.19	13	23.51	12.74	4.70
Fever + Cough	11	10.98	7.75	0.00	10	8.07	6.37	0.46	10	11.95	9.80	0.32
Fever + Fatigue	25	26.56	17.60	0.09	31	19.53	19.74	6.74	19	28.91	18.63	3.40
Cough + Fatigue	11	11.33	7.75	0.01	9	8.33	5.73	0.05	12	12.33	11.77	0.01
Fever + Cough + Fatigue	38	35.77	26.76	0.14	37	26.30	23.57	4.35	26	38.93	25.49	4.30

χ^2^_(6)_ = 59.20 *p* < 0.01 Post ad hoc estimation Likelihood-ratio χ^2^_(6)_ = 52.31 *p* < 0.01.

**Table 5:** Precautionary measures taken by dentists that continued to work after the outbreak of COVID-19.

	Item	*n* (%)
Before patient arrival	Phone Triage	2542 (82.37)
	Spaced appointments as not saturate the waiting room	2656 (86.07)
	Deferring therapies in elderly patients, or with systemic diseases	1912 (61.96)
	Detecting body temperature of all co-workers and leave those with a temperature above 37.5 °C.	656 (21.26)
In the waiting room	Disinfection of pushbuttons, POS, chairs, several times a day	2525 (81.82)
	Verify the patient’s current health status on access	2568 (83.21)
	Detecting the patient’s body temperature	725 (23.49)
	Washing the patient’s hands	2413 (78.19)
	Space of at least one meter between patients	2312 (74.92)
	Mask for the patient	1011 (32.76)
	Frequent ventilation of waiting rooms	2746 (88.98)
	Removal of magazines and books from the waiting area	2418 (78.35)
	Storage of coats, bags, and other items outside the operating area	2103 (68.15)
In the operating room	Pre-operative rinse with mouthwash containing 1% hydrogen peroxide	813 (26.34)
	Pre-operative rinse with mouthwash containing chlorhexidine 0.12–0.2%	1658 (53.73)
	Pre-operative rinse with mouthwash containing 0.2–1% iodopovidone	251 (8.13)
	Pre-operative rinse with mouthwash containing alcohol and essential oils	190 (6.16)
	Pre-operative rinse with mouthwash with Cetylpyridinium chloride at 0.05–0.10%	86 (2.79)
	Rinse with diluted mouthwash	112 (3.63)
	Ventilation of the operating area for at least 10 min after each patient	2379 (77.09)
	Disinfection of surfaces with 70% ethyl alcohol	1264 (40.96)
	Disinfection of surfaces with 0.5% sodium hypochlorite	611 (19.80)
	Disinfection of surfaces with usual disinfectant with other active ingredients	1875 (60.76)
	Washing operators’ hands before and after each procedure	2828 (91.64)
	Removal of all disposable protective devices and disinfection of devices	2484 (80.49)

**Table 6:** Precautionary measures against COVID-19 stratified by areas with different prevalence of the disease. The items with 80% or more positive replies were used. Percentages were calculated per column.

Answers	Milan Area				High Prevalence Area				Low Prevalence Area			
	OF	EF	%	Cχ^2^	OF	EF	%	Cχ^2^	OF	EF	%	Cχ^2^
Phone triage χ^2^_(2)_ = 11.41 *p* < 0.01 Post ad hoc estimation Likelihood-ratio χ^2^_(2)_ = 11.44 *p* < 0.01												
No	185	344.3	17.57	3.90	126	252.7	15.91	0.04	233	372.9	18.78	4.27
Yes	868	1092.98	82.43	1.51	666	803.58	84.09	0.02	1008	958.1	81.22	1.66
Appointments delayed so to not saturate the waiting room χ^2^_(2)_ = 6.78 *p* = 0.03 Post ad hoc estimation Likelihood-ratio χ^2^_(2)_ = 6.84 *p* = 0.03												
No	96	305.1	9.12	1.05	123	223.7	15.53	0.92	211	330.2	17.00	3.14
Yes	957	923.9	90.88	0.35	669	677.3	84.47	0.30	1030	998.8	83.00	1.04
Disinfection of pushbuttons, POS, chairs, several times a day χ^2^_(2)_ = 8.04 *p* = 0.02 Post ad hoc estimation Likelihood-ratio χ^2^_(2)_ = 8.10 *p* = 0.02												
No	107	352.61	10.16	1.55	202	258.79	25.50	0.67	212	381.59	17.08	3.50
Yes	946	876.38	89.24	0.62	590	643.20	74.50	0.27	1029	948.41	82.92	1.41
Verify the patient’s current health status on access χ^2^_(2 )_= 8.79 *p* = 0.01 Post ad hoc estimation Likelihood-ratio χ^2^_(2)_ = 8.56 *p* = 0.01												
No	161	336.28	15.29	1.81	89	246.81	21.60	0.67	268	363.91	16.78	3.75
Yes	892	892.72	84.71	0.69	703	655.19	78.40	0.23	973	966.09	83.22	1.41
Frequent ventilation of waiting rooms χ^2^_(2)_ = 5.61 *p* = 0.06 Post ad hoc estimation Likelihood-ratio χ^2^_(2)_ = 5.62 *p* = 0.06												
No	299	275.12	24.33	2.07	204	201.92	22.62	0.02	272	297.95	20.44	2.26
Yes	930	953.87	75.67	0.60	698	700.08	77.38	0.01	1059	1033.04	79.56	0.65
Washing operators’ hands before and after each procedure χ^2^_(2)_ = 9.21 *p* = 0.01 Post ad hoc estimation Likelihood-ratio χ^2^_(2)_ = 9.32 *p* < 0.01												
No	262	246.15	21.32	1.02	199	180.46	22.09	1.91	232	266.38	17.44	4.44
Yes	967	982.45	78.68	0.26	702	720.54	77.91	0.48	1098	1063.62	82.56	1.11
Removal of all disposable protective devices and disinfection of devices χ ^2^_(2)_ = 9.09 *p* = 0.01 Post ad hoc estimation Likelihood-ratio χ^2^_(2)_ = 9.17 *p* = 0.01												
No	392	365.86	21.32	1.87	281	267.92	22.09	0.64	357	396.22	17.44	3.88
Yes	837	863.14	78.68	0.79	619	632.08	77.91	0.27	974	934.77	82.56	1.65

**Table 7:** Personal protective equipment (PPE) and devices adopted by the dentists.

Items	*n* (%)
Surgical mask	2386 (74.56)
FFP2 or FFP3 facial filters	1755 (54.84)
Disposable headset	2040 (63.75)
Sterile microfiber disposable gown	675 (21.09)

**Table 8:** Perception of risk related to COVID-19.

Items as *n* (%)			
Do you believe that the infection by SARS-CoV-2 is a risk for the dentist?			
Very unlikely	Unlikely	Likely	Very likely
107 (3.11)	121 (3.52)	993 (28.91)	2214 (64.50)
How sure are you that you can avoid being infected by SARS-CoV-2 during work?			
Not confident	A bit confident	Enough confident	Confident
1275 (37.20)	966 (28.19)	1113 (32.48)	73 (2.13)
In a health emergency situation such as the current one, do you believe that the risk of infection transmission in the dental practice is:			
Higher than the risk run in a supermarket	Comparable to the risk run in a supermarket	Less than the risk run in a supermarket	
2349 (68.50)	405 (11.81)	675 (19.69)	

**Table 9:** Risk perception of COVID-19 stratified by areas with different prevalence of COVID-19. Percentages were calculated per column.

Answers	Milan Area				High Prevalence Area				Low Prevalence Area			
	OF	EF	%	OF	EF	%	OF	EF	%	OF	EF	%
Do you believe that the infection by SARS-CoV-2 is a risk for the dentist? χ^2^_(6)_ = 13.54 *p* = 0.03 Post ad hoc estimation Likelihood-ratio χ^2^_(6)_ = 13.67 *p* = 0.03												
Very unlikely	48	40.97	3.74	1.21	22	30.40	2.51	2.32	46	44.64	3.57	0.04
Unlikely	38	37.08	2.96	0.02	29	27.51	3.31	0.08	38	40.41	2.95	0.14
Likely	311	344.67	24.26	3.29	289	255.74	32.95	4.33	376	375.59	29.19	0.00
Very likely	785	759.28	61.24	0.87	537	563.36	61.23	1.23	828	827.37	64.29	0.00
How sure are you that you can avoid becoming infected with SARS-CoV-2 during work? χ^2^_(6)_ = 17.91 *p* < 0.01 Post ad hoc estimation Likelihood-ratio χ^2^_(6)_ = 17.99 *p* < 0.01												
Not confident	482	436.53	40.95	4.74	292	325.26	32.30	3.40	464	476.21	36.13	0.31
Enough confident	321	334.62	27.27	0.56	278	249.33	31.70	3.30	350	365.04	27.25	0.62
A bit confident	349	380.46	29.66	2.60	286	283.49	32.61	0.02	444	415.05	34.60	2.02
Confident	25	25.38	2.12	0.01	21	18.92	2.39	0.23	26	27.70	2.02	0.10
In a health emergency situation such as the current one, do you believe that the risk of infection transmission in the dental practice, compared to that run in a supermarket, is χ^2^_(4)_ = 16.08 *p* < 0.01 Post ad hoc estimation Likelihood-ratio χ^2^_(42)_ = 16.04 *p* < 0.01												
Lower	211	232.78	17.91	2.04	200	173.30	22.80	4.11	249	253.92	19.38	0.09
Comparable	121	140.73	10.27	2.77	119	104.77	13.57	1.93	159	153.51	12.37	0.20
Higher	846	804.05	71.82	2.14	558	598.93	63.63	2.80	877	877.57	68.25	0.01

## Supplementary Materials

The following are available online at https://www.mdpi.com/1660-4601/17/11/3835/s1, Table S1: Row data.

## Abbreviation

MERS-CoV	Middle East Respiratory Syndrome MERS-CoV
SARS-CoV	Severe Acute Respiratory Syndrome
COVID-19	Coronavirus Disease
POS	Point of sale
PPE	Personal Protective Equipment
NHS	National Health System
